# Building Resources in Caregivers: Feasibility of a Brief Writing Intervention to Increase Benefit Finding in Caregivers

**DOI:** 10.1111/aphw.12195

**Published:** 2020-02-05

**Authors:** Stephen Gallagher, Liam O’Sullivan, Zoe Hughes, Brenda H. O’Connell

**Affiliations:** ^1^ University of Limerick Limerick Ireland; ^2^ Health Research Institute University of Limerick Limerick Ireland; ^3^ Care Alliance Ireland Dublin Ireland; ^4^ University College Cork Cork Ireland; ^5^ National University of Ireland Maynooth Maynooth Ireland

**Keywords:** benefit finding, caregiver, expressive writing, family‐carers, feasibility, intervention

## Abstract

The Building Resources in Caregivers (BRiC) is a pilot feasibility trial that compared the effects of a 2‐week benefit finding writing expressive intervention to a control intervention, who wrote about the weather. Caregivers completed primary (benefit finding) and secondary (quality of life, depression and anxiety) outcome measures at pre (t1), immediately post‐test (t2) and 1 month later (t3). They also completed measures relating to trial feasibility, difficulty, and acceptance. Using complete case analysis only, analysis revealed no effect of the intervention for primary or secondary outcomes. Despite this, there were no differences between the intervention and control groups on key feasibility measures. Caregivers in the control condition were less likely to recommend this to other caregivers. Moreover, qualitative commentary provided by caregivers suggested that not everyone enjoyed the writing, some found it stressful, offering up some explanation for our findings. Our pilot trial suggests that any future benefit‐finding writing intervention would require several procedure modifications including tailoring to a specific cohort of caregivers, in particular those who like writing, before it has some utility as a psychosocial intervention.

## Introduction

Associations between caregiving and negative effects on caregivers’ health have been widely researched (Goode, Haley, Roth, & Ford, 1998; Grunfeld et al., 2004; Thomas et al., 2006). These effects have been linked to increased physical health problems (Gallagher & Whiteley, 2013; Pinquart & Sörensen, 2003), poor sleep quality (Mui, 1995), and higher levels of depression than in the non‐caregiver population, ranging from 22 per cent to 30 per cent (Schulz, O’Brien, Bookwala, & Fleissner, 2010). However, while the negative consequences are well established, it has been noted that they are not the only outcomes of research interest. As Walker et al. (1996) commented, “the disproportionate emphasis in the literature on burden and depression has deflected our attention from significant positive outcomes” (p. 139), a sentiment echoed by Chen and Greenburg (2004). In response, subsequent research efforts have attempted to elucidate psychological constructs associated with positive outcomes such as benefit finding (BF) and their influence on the quality of life (QoL) in caregiver populations (Brand, Barry, & Gallagher, 2014).

Benefit finding is a process by which people perceive positive growth from stressful and traumatic experiences (Linley & Joseph, 2004). It has been defined by Helgeson, Reynolds, and Tomich (2003, p. 797) as “the positive effects that result from a traumatic event”, and they go on to suggest that people who suffer a traumatic event may engage in benefit finding as a cognitive strategy for coping with distress in the short term, but that it may also be a reliable measure of actual positive growth in the longer term. In the caregiving context, the available literature has identified benefit finding as a variable that has direct influences on the perception of QoL, and these influences are known to help manage the negative outcomes associated with caregiving duties. For example, Cohen (2002) found that over 70 per cent of caregivers in their sample (*n* = 289) derived positive experiences in the areas of companionship and a sense that being a caregiver was rewarding. More recently, the association between benefit finding and caregiver QoL was found to be mediated by social support (Brand et al., 2014), with those high in benefit finding also reporting higher social support and better QoL. Given the importance of benefit finding for caregiver health outcomes, it is hardly surprising that it has become the target of interventions for caregivers (Brand et al., 2014; Cheng, Lau, & Mak, 2012).

Researchers have suggested that caregivers may engage in BF as a cognitive strategy for coping with stress (Brand et al., 2014; Slattery, McMahon, & Gallagher, 2017). Moreover, as part of this cognitive strategy their view of themselves, others, and their place in the world is transformed to produce positive growth and psychological change during or after traumatic events through a process of cognitive restructuring (Cheng et al., 2014; Tedeschi & Calhoun, 2004). In fact, benefit finding as proposed by Affleck and Tennen (1996) facilitates cognitive restructuring whereby people view stressors in a positive light. Moreover, given that this has been associated with improvements in mood after stressful events (Folkman & Moscowich, 2000), one can see why it has become the focus of intervention research (Cheng et al., 2014). Here we report on the findings of a brief pilot study to test the feasibility and acceptability of a brief BF writing intervention for caregivers. The protocol of this intervention has been published elsewhere (Brand et al., 2014).

### Writing as a Therapy

The use of writing in a therapeutic manner derives from psychotherapeutic and positive psychology traditions characterised by interpersonal disclosure, which can include identifying, labelling, and disclosing emotional experiences in the case of the former (Smyth & Helm, 2003) and attending to signature strengths and virtues in the case of the latter (O’Connell, O’Shea, & Gallagher, 2018; Seligman, Steen, Park, & Peterson, 2005). Individuals taking part in these interventions are typically instructed to write without regard to spelling, style or grammar, and are informed that their written work or narratives will remain confidential. Depending on the research question or topic under investigation, the time devoted to writing can vary, with some taking a few minutes every day while others suggest writing for 15–30 min each day over several consecutive days (Merz & Malcarne, 2014; Zachariae & O’Toole, 2015). Although initial studies asked participants to write about traumatic events as a way to facilitate cognitive processing and encourage recovery from the event (Frattaroli, 2006; Pennebaker, 1997), other studies have modified the writing instructions given to participants to focus on the positive experiences associated with these events (Stanton et al., 2002). This shift has been driven primarily by researchers interested in testing whether writing about the positive experiences, rather than the negative, can produce health benefits (Stanton et al., 2002). One study adopting this approach found that undergraduate students who wrote about the perceived benefits of personal trauma made fewer visits to the university health centre in the months following writing compared to a control group (King & Miner, 2002). In the caring context, parents caring for children with autism who wrote about the benefits of caring for these children were found to be less anxious after the writing exercises compared to control parents (Lovell, Moss, & Wetherell, 2016).

Due to the nature of caregiving, a lack of time for oneself, inability to meet their own personal needs (Grant & Davis, 1997), respite constraints (Pasacreta & McCorkle, 1999) inaccessibility to the research site, and mistrust of researchers (Dowling & Wiener, 1997; Gallagher‐Thompson et al., 2003; Lampley‐Dallas, 2002; Moreno‐John et al., 2004), we aimed to make our intervention as brief as possible to take into account caregiver needs. Hence, to circumvent these obstacles, we adopted an e‐health and positive psychology weekly diary writing approach as this also has the potential to remove the time and space barrier between caregivers and therapists. Based on the above research, in a randomised controlled trial, we aimed to test the feasibility and acceptability of a brief BF writing intervention using the internet and included BF as a primary outcome and QoL and psychological well‐being as secondary outcomes. We hypothesised that caregivers in the intervention arm would have increased levels of BF, better QoL and well‐being afterwards.

## Methods

### Participants

**Figure 1:**
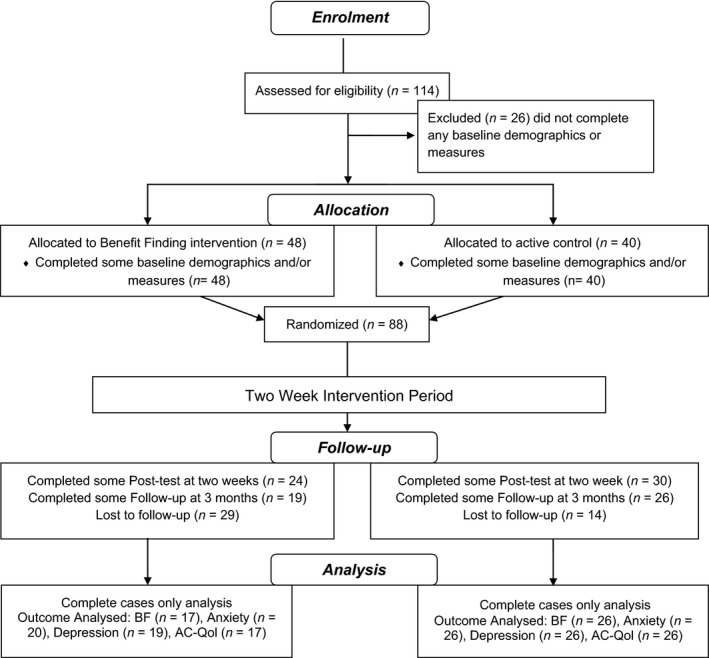
Flow of participants through trial stages adapted from CONSORT 2010 Flow Diagram (Schulz et al., 2010).

Caregivers were recruited through our NGO collaborators (Care Alliance Ireland and the Carer’s Association of Ireland) using their social media pages in the build‐up to National Carers Week in May/June 2015. A donation of €2,000 was made to the Carer’s Association for assisting with recruitment. Inclusion criteria were primary caregivers in the Republic of Ireland aged over 18 years, currently providing care to one or more persons. Those excluded from the proposed study were professional caregivers such as those who are in the employment of institutions of care or home care provision companies and foster carers. In terms of sample size and power, calculations were based on previous research (Cheng et al., 2014). For a medium effect size of *F* = .25, *p* < .05, and power of .8, the total sample size required for this pilot was 86, with 43 in each trial arm (calculated with G*Power Version 3.9). Further, assuming a dropout rate of approximately 10.5 per cent, we aimed to recruit a sample size of 96 caregivers to provide data for the final analysis. Figure 1 has the CONSORT table with participant recruitment. Summary sociodemographic characteristics of participants by treatment group are presented in Table 1. The study was approved by the local university research ethics committee and each participant gave informed online consent.

**Table 1:** Sample Characteristics and Baseline Differences across Conditions

	Benefit finding (*n* = 48)		Control (*n* = 40)		Between‐group differences
	*M*	*SD*	*M*	*SD*	*p*
Caregiver age	47.62	9.04	48.27	8.82	.733
Age of care recipient	49.62	30.45	51.67	30.2	.759
Hours sleep per night	5.85	1.22	5.40	1.47	.127
Years caregiving	10.49	8.60	10.73	9.30	.904

	*n*	%	*n*	%	
Sex (female)	44	91.7	39	97.5	.239
Married/living together	26	54.2	24	60.0	.775
Ethnicity (white Irish)	41	85.4	38	95	.441
Education					
Some or all of secondary school	16	33.3	9	22.5	.241
Additional training (e.g. FAS)	17	35.4	11	27.5	
Undergraduate degree	12	25.0	13	32.5	
Postgraduate degree	3	6.3	7	17.5	
Household income					
€0–€20,000	20	41.7	14	35.0	.478
€20,001–€40,000	22	45.8	21	52.5	
€40,001–€60,000	4	8.3	5	12.5	
€60,001–€80,000	2	4.2	–	–	
Outside work per week					
0 hours	31	64.6	27	67.5	.147
1–15 hours	11	22.9	7	17.5	
16–24 hours	–	–	2	5.0	
25–34 hours	–	–	3	7.5	
35–39 hours	3	6.3	1	2.5	
≥40 hours	3	6.3	–	–	
Hours caregiving per week (>71)	25	52.1	31	77.5	.316

### Study Design

This double blind RCT parallel group trial design adhered to the CONSORT guidelines (Turner et al., 2012) and had three measurement periods (T1—baseline, T2—immediately after intervention, and T3—3 months later). Group (intervention vs. control) was our independent variable and our primary outcome was BF. Parallel to these outcomes we were interested in looking at acceptability and difficulty with the writing tasks (Peterkin & Prettyman, 2009), assessed levels of well‐being, and QoL.

### Measures

Caregiver and care‐recipient sociodemographics including age, gender, socioeconomic indices, and health‐related variables such as illness types and hours spent caring were assessed by questions created in‐house.

#### Primary Outcome

Benefit finding was measured using the 17‐item Benefit Finding Scale (BFS; Antoni et al., 2001). Examples of items include, “Has led me to be more accepting of things”, and “Has helped me to take things as they come”, which are scored using a 5‐point Likert type scale ranging from (1) “not at all” to (5) “extremely”, where higher values indicate a higher degree of benefit finding. The scale has excellent reliability (Cronbach’s α = .91) (Kim et al., 2007; Urcuyo et al., 2005. We also altered the wording of each item to reflect current feelings (e.g. “I am more accepting of things”, “I take things as they come”) so that we could capture change following the intervention. For T2 and T3 follow‐up measures of benefit finding, the stem was be changed to “After taking part in this writing exercise how much have the following changed for you? As a caregiver, I feel that …”, with the same 17 original items used.

#### Secondary Outcomes

Caregiver QoL was captured using the 40‐item Adult Carer Quality of Life Questionnaire (AC‐QoL) (Joseph et al., 2012). The Hospital and Anxiety Depression Scale (HADS; Zigmond & Snaith, 1983) assessed symptoms of anxiety and depression.

#### Fidelity, Implementation, and Adherence

The writing instructions are a core component of the proposed intervention. In order to ensure as high a level of adherence to fidelity criteria associated with the main outcome measure (BF) as possible, the writing instructions for both the control and intervention groups were constructed in such a way as to induce an equal chance of perceiving benefit. Additionally, to assess the level of expectation of treatment efficacy across both groups of perceiving benefit, prior to random allocation, all participants were asked to answer the following question: “How well do you expect to feel after taking part in the writing activities?”, on a Likert type scale ranging from 1 (*Not at all*) to 4 (*Very well*). We expected both groups to have a similar level of expectation to benefit from the writing exercise. Moreover, to assess participants’ difficulty and acceptability of the writing exercise at T2, participants were asked a series of questions rated on 5‐point Likert type response scales. These included, “How difficult did you find the writing activities to do?” (1 = *Not at all difficult*, to 5 = *Very difficult*); “How disruptive of your time did you find the writing activities?” (1 = *Not at all disruptive*, to 5 = *Very disruptive*); “How acceptable were the writing activities for you?” (1 = *Not at all acceptable*, to 5 = *Very acceptable*); “How likely would you be to recommend this type of writing activity to other carers?” (1 = *Not at all likely*, to 5 = *Very likely*). Further, to improve adherence, caregivers received an email prompt each week to remind them to write in their diaries. Finally, also included was a free form text box where caregivers could respond freely to the question, “If there is anything that we may have forgotten to ask about your experiences of writing then feel free to write in the space provided.”

#### Intervention

The proposed intervention was informed by prior work in the BF interventions literature (Cheng et al., 2014; Henry et al., 2010) which found that cognitive approaches that focused on increasing benefit finding among caregivers had superior results in reducing depression over psychoeducational approaches attempting to achieve the same goal. Caregivers were instructed to write about their thoughts and feelings in a diary/personal notebook focusing on the benefits of caring, to consider improved social relationships, the appreciation of life and loved ones, and think about the positive consequences with respect to these. To better meet the needs of the caregivers in our study (e.g. time pressures, sensitivity) we did not ask for diaries to be returned. These activities were to be done three times a week for 2 weeks. The control group were asked to write about the weather that day for the same number of days.

The intervention group writing instruction read as follows:

Writing is a great way to reflect on your life in general and on the roles we have in life, helping to look back and focus on the good things in our lives. These things can be big and small. For the next two weeks, three times a week on the days of your choosing we encourage you to really let go, explore your innermost thoughts and feelings about the benefits of providing care to your loved one and write these thoughts down. Examples could include writing about becoming closer to your loved one, focusing on the things your loved one is able to do rather than what they cannot do, no matter how big or small this is, feeling needed, more empathetic, accepting, compassionate, new relationships with others, your loved one or family members; perhaps seeing your life in a different way—more positive. It could also be about you and how satisfied you are as a carer moving forward and learning how your priorities have changed. These are just examples, but you may have other benefits that you would like to talk about regarding your caring role, no matter how big or small they may be. Although when you write the sentences they can be as long or as short as you like but try and aim for about 3 or 4 sentences at least.

The control group writing instruction read as follows:

Writing is a good way of getting us to reflect a bit better and it may help improve our well‐being. For the next two weeks, on just six days of your choice (3 days each week), write in a copybook or a personal diary, a number of things about the weather on each day. Although when you write the sentences they can be as long or as short as you like but try and aim for about 3 or 4 sentences at least.

#### Procedure

After providing informed consent, caregivers were randomly allocated using the random allocation feature of survey QuestBack™ software to the intervention or control condition. Baseline measures were then completed at T1 (e.g. demographics, care recipients details, primary and other outcome measures); after the 2‐week writing stage was complete, participants were sent an email with a link to complete the post‐intervention (T2), and 3‐month follow‐up (T3) assessment, with outcomes as previously administered at baseline.

### Statistical Analysis

Prior to formal statistical analysis, data were checked for assumption of fit and suitable descriptive analyses were conducted on the baseline characteristics. To test the effectiveness of the intervention versus the active control on the primary outcomes benefit finding, and secondary outcomes quality of life, anxiety, and depression, across each follow‐up, mixed between‐within analyses of variance were conducted using complete cases only. Further, based on previous work (O’Connell, O’Shea, & Gallagher, 2016), high attrition rates were predicted, so sensitivity analysis was conducted using linear mixed modelling where an intention‐to‐treat analysis strategy was employed with inclusion of all randomised participants. This includes all available data, and maximum likelihood estimations were implemented to handle missing data. Finally, key qualitative comments are presented as a way of informing our results and future study designs.

## Results

### Preliminary Analysis

#### Attrition

Figure 1 displays the participant flow through each stage of the study (complete cases only) following the CONSORT guidelines (Schulz et al., 2010). Of the 88 caregivers who completed baseline questionnaires (at least in part), 45 (51.1%) completed both the post‐intervention and follow‐up assessments. Caregivers who dropped out of the study (*n* = 43) did not differ from those who completed all three parts (*n* = 45) with respect to demographic characteristics (i.e. sex, χ^2^(1) = .167, *p* = .68; age; *F*(1, 85) = .35, *p* = .556); baseline BF scores, *F*(1, 79) = .059, *p* = .809; depression scores, *F*(1, 82) = .034, *p* = .854; anxiety scores, *F*(1, 82) = .045, *p* = .832; and AC‐QoL, *F*(1, 78) = .038, *p* = .847. Dropout was, however, associated with increased likelihood of being in the intervention group rather than the control group, χ^2^(1) = 5.64, *p* = .018. Table 1 reports participant characteristics.

#### Randomisation and Adherence

Tests of baseline homogeneity were conducted between the intervention and control groups and no significant differences were found in demographic and socioeconomic indices (see Table 1), or in primary and some secondary outcomes of interest; BF, *F*(1, 79) = .376, *p* = .541; depression, *F*(1, 82) = 1.28, *p* = .262; or AC‐QoL, *F*(1, 78) = .858, *p* = .357; however, differences were found for baseline anxiety, *F*(1, 82) = 5.24, *p* = .025, with caregivers in the control group reporting higher levels of anxiety at baseline (*M* = 12.10) than the intervention group (*M* = 10.43).

Reported adherence to the activities at post‐intervention did not differ significantly between the conditions (*p* = .99), with a mean usage of 3.6 days across conditions (*SD* = 1.98). Caregivers had similar beliefs about the treatment efficacy (*p* = .351) regardless of whether they were in the control (*M* = 2.65, *SD* = .95) or the intervention group (*M* = 2.84, *SD* = .86). Descriptive characteristics across time and treatment groups are presented in Table 2.

**Table 2:** Outcome Characteristics and Differences in Groups across Time (GLM mixed) for Complete Cases

	Benefit finding						Control											
	Time 1 (*n* = 48)		Time 2 (*n* = 24)		Time 3 (*n* = 19)		Time 1 (*n* = 40)		Time 2 (*n* = 20)		Time 3 (*n* = 24)		Effect of time			Interaction effect		
	*M*	*SD*	*M*	*SD*	*M*	*SD*	*M*	*SD*	*M*	*SD*	*M*	*SD*	*F*	*df*	*p*	*F*	*df*	*p*
Benefit Finding	3.82	.56	3.68	.82	3.43	1.23	3.75	.56	3.56	.78	3.36	.90	2.63	2.82	.078	.478	2.82	.622
Depression	7.79	4.10	6.96	4.59	7.20	4.82	8.85	4.44	9.23	4.37	8.77	4.26	.521	2.86	.596	.772	2.86	.456
Anxiety^a^	10.43	3.37	8.67	4.58	9.60	5.10	12.1	3.24	11.47	3.94	11.61	4.29	.104	1.97, 84.86	.748	.313	1.97, 84.86	.579
Quality of life	2.65	.42	2.63	.26	2.68	.48	2.57	.34	2.54	.30	2.52	.40	.558	2.82	.499	.451	2.82	.551

Note

Assumption of sphericity was violated for quality of life; thus, Greenhouse‐Geisser correction is reported.

a As anxiety scores differed at baseline between conditions, this was controlled for by entering baseline anxiety as a covariate and time 2 anxiety scores and time 3 anxiety scores as the within‐subjects factors in the GLM mixed analysis. Controlling for baseline anxiety in all other GLM mixed analysis did not alter the null findings.

#### The Effect of Treatment on Psychosocial Outcomes Over Time

Using complete case analysis only, a mixed between‐within analysis of variance revealed no main effect for time and no Treatment × Time interaction, for BF, anxiety, depression, or AC‐QoL (all *p *> .05), suggesting that there was no effect of the intervention on any of the outcomes over time compared to the control group (see Table 2). To account for the missing data, multilevel modelling was run on all available data using an intention‐to‐treat approach. The null findings found with the complete‐cases only analysis were supported by the Linear Mixed Models which indicated that trends in all outcomes (BF, anxiety, depression, and AC‐QoL) did not vary significantly over time between the two treatments (see Table 3). For benefit finding as outcome, a basic model, in which all parameters were fixed, produced a −2LL of 465.85. Including a random intercept to the model significantly improved the overall fit of the model, −2LL = 431.06, χ^2^(1) = 34.79, *p* < .01, and including a random intercept and slope to the model significantly improved the overall fit of the model, −2LL = 397.65, χ^2^(1) = 33.41, *p* < .01. However, allowing covariance between the random slopes and random intercepts did not significantly improve model fit (−2LL = 395.06, χ^2^(1) = 2.59, *p *> .05). Therefore, the model with random intercepts and slopes and seven parameters—four fixed (intercept, time, intervention group, and time × intervention), two random effects (intercept and time), and one estimated residual variance—was used for the multilevel analysis on benefit finding. For depression as outcome, a basic model in which all parameters were fixed produced a −2LL of 1,138.09. Including a random intercept to the model significantly improved the overall fit of the model, −2LL = 1036.98, χ^2^(1) = 101.11, *p* < .01. However, inclusion of a random slope did not significantly improve model fit (−2LL = 1035.9, χ^2^(1) = 1.08, *p *> .05). Therefore, the model with random intercepts and six parameters—four fixed (intercept, time, intervention group, and time × intervention), one random effect (intercept), and one estimated residual variance—was used for the multilevel analysis on depression. For anxiety as outcome, a basic model, in which all parameters were fixed, produced a −2LL of 1,105.07. Including a random intercept to the model significantly improved the overall fit of the model, −2LL = 1,042.31, χ^2^(1) = 62.76, *p* < .01, and including a random intercept and slope to the model significantly improved the overall fit of the model, −2LL = 1,032.21, χ^2^(1) = 10.1, *p* < .01. However, allowing covariance between the random slopes and random intercepts did not significantly improve model fit (−2LL = 1031.23, χ^2^(1) = 0.98, *p *> .05). Therefore, the model with random intercepts and slopes and seven parameters—four fixed (intercept, time, intervention group, and time × intervention), two random effects (intercept and time), and one estimated residual variance—was used for the multilevel analysis on anxiety. For QoL as outcome, a basic model in which all parameters were fixed produced a −2LL of 168.35. Including a random intercept to the model significantly improved the overall fit of the model, −2LL = 132.65, χ^2^(1) = 35.7, *p* < .01. However, inclusion of a random slope did not significantly improve model fit (−2LL = 131.37, χ^2^(1) = 1.28, *p *> .05). Therefore, the model with random intercepts and six parameters—four fixed (intercept, time, intervention group, and time × intervention), one random effect (intercept), and one estimated residual variance—was used for the multilevel analysis on QoL.

**Table 3:** Linear Mixed Modelling Fixed Effects of Treatment by Time

	Model parameters	Model fit (−*2LL*)	Fixed effects: treatment × time				
			Estimate	*SE*	*t*	*p*	95% CI
Benefit finding	7, random intercepts & slopes	397.65	−.03	.11	−.236	.814	−.25, .20
Depression	6, random intercepts	1036.98	.2	.29	.506	.613	−.58, .98
Anxiety	7, random intercepts & slopes	1032.21	.42	.49	.871	.386	−.54, 1.39
Quality of life	6, random intercepts	132.65	−.04	.05	−.918	.361	−.13, .05

Note

Models were fitted with a variance components covariance structure. For depression and quality of life as outcomes, there was no significant variability in slopes and model fit did not significantly improve when allowed to be at random, so best fit model is reported.

#### Feasibility

Caregivers in the intervention condition (*M* = 3.25, *SD* = 1.19) did not differ significantly from those in the control condition (*M* = 3.23, *SD* = 1.17) in perceived difficulty of the activity, *F*(1, 1) =0.003, *p* = .9. There were also no statistically significant differences between the intervention condition (*M* = 3.96, *SD* = 1.16) and the control condition (*M* = 3.8, *SD* = 1.06) on how disruptive they found the activities, *F*(1, 1) = 0.273, *p* = .605. Although many participants reported that the tasks were disruptive (*Median* = 4), at the same time caregivers reported that they were acceptable (*Md* = 4). Levels of acceptability did not differ significantly between those in the intervention condition (*M* = 4.17, *SD* = .70) and those in the control condition (*M* = 3.67, *SD* = 1.12). Of those who responded (*n* = 54), only 12 caregivers stated that they were not, or not at all, likely to recommend this type of writing activity to other carers, while 27 stated that they were either likely or very likely to recommend this activity to other carers. Caregivers in the intervention condition (*M* = 3.88, *SD* = 1.08) were more likely to recommend this to others than those in the control condition (*M* = 3.0, *SD* = 1.29), *F*(1, 1) =7.11, *p* = .01.

Our qualitative comments add more detail to the feasibility data above. For example, some caregivers in the experimental group reported that “it was harder to do the more it went on” or that “it was harder on days that were more stressed” and “a new habit is hard to establish”. These comments may help explain why attrition was higher in this group relative to the control group. However, some carers in the control group also found their writing topic to be a bit boring: “The weather topic was very Irish! Having a more interesting topic to write on would have been better.” While others in the same group said: “I felt the writing activity was another stress in my life” or “I found it difficult to write and I lost concentration a lot”. Thus, while it was acceptable for some, others found it cumbersome and boring with the opposite effect happening—adding stress to their lives. Nonetheless, this was not always the case, with some positive comments from those in the experimental and the control group showing the merit of such approaches. For example, some in the experimental group wrote, “it’s great for helping me find perspective” and “it made me realise the closeness I have with my mum and how I appreciate the little things like her cheeky grin or a gentle touch”. While for others, it was easier to do as they are used to writing as a profession: “I am a writer so I did enjoy it”. One in the control group said that “personally it gave me something external to think about and appreciate what was going on around me!” and another said “I like the writing it makes me think about something unrelated to caring”. Thus, these latter comments in the control group, while they demonstrate that it is acceptable for some people, they also suggest that the activity served as a distraction from their caring duties, and perhaps offering some therapeutic value. Taken as a whole, the qualitative comments, however, do highlight that it is not a one size fits all approach, and that while there are merits to writing interventions for carers, they also suggest that for some it is not a suitable activity and may offer non‐tangible benefit, and even add to their stress. However, for others, in particular those who may enjoy writing, it had clear benefits and afforded them the opportunity to reappraise their situation in a positive manner.

## Discussion

This BF writing exercise randomised controlled pilot trial, while it did not improve our main outcome variables, was deemed feasible and acceptable by our caregiver sample. This was pertinent to us as there are multiple obstacles for caregivers to take part in research interventions and we aimed to see whether our intervention would address some of these challenges. Despite its proposed promise, the results from the trial on the main outcome variables will require a rethink on several methodological areas before moving to a larger, adequately powered study in order to reach any conclusion regarding its clinical utility. Even though the brief writing exercise programmes we tested were similar on levels of adherence, that is, mean of 3.6 days over the 2‐week period, treatment expectations prior to taking part, acceptability, difficulty and disruption to their daily activities, the brevity, focus and number of sessions required of the writing sessions may need to be reconsidered in future trials. Further, the caregivers in the control group were less likely to recommend this type of intervention to others, implying that there were issues with writing about the weather. Moreover, some of the qualitative responses provide some food for thought on how the writing intervention could be improved but also for whom it may be best suited.

In terms of BF interventions to improve psychological outcomes, recent studies have had a more structured and longer intervention period to assist with cognitive restructuring. For example, Cheng et al (2014) asked caregivers to attend a face‐to‐face session for 2 hours per week for 8 weeks; and this resulted in reduced depression and caregiver burden after the intervention. Thus, given that our trial was only 2 weeks—and only required caregivers to write 3 times per week with no time limit prescribed for actual writing sessions—it may be that our trial was too brief to allow positive reappraisal to occur at any meaningful level. In addition, recent meta‐analyses on writing interventions have also found them to be ineffective in improving psychological outcomes, especially if they are brief (Reinhold, Bürkner, & Holling, 2018). In fact, these authors suggest that these writing interventions should be longer, more directed, and with additional therapeutic support. In other brief writing studies, Mackenzie, Wiprzycka, Hasher and Goldstein (2007) found no improvements in psychological well‐being for expressive writing after a 2‐week period. Further, not only is the duration of the intervention important, other reviews have suggested that there are moderators of writing interventions including extended duration and spacing of the writing exercises (Travagin, Margola, & Revenson, 2015), and social constraints, with those low on emotional support doing better (Merz, Fox, & Malcarne, 2014).

Our qualitative comments also provide some insight and extend on some of the moderating factors identified above. Our control exercise of writing about the weather, while supposed to be neutral, was seen as boring; thus a more engaging topic would need to be adopted in future trials. In addition, some of the caregivers found it harder to keep up the writing as it went on, so perhaps reducing the weekly sessions but over longer duration would have been more beneficial—which ties into the spacing issue identified in previous reviews (Travagin et al., 2015). Further, while the focus of the writing task was on the benefits of caring, it must be acknowledged that there are day‐to‐day stressors and strains that would affect this; therefore, a reframing of the benefit‐finding intervention to take account of this may be more fruitful. Moreover, not all caregivers may be motivated to see the positive and could be at a different stage and may not see it as a support. Indeed, along this line, what was very evident in the qualitative comments, across both groups, is that writing interventions do not suit all caregivers. In particular, some caregivers found writing stressful or cumbersome, while others, especially those who liked writing and were able to express themselves, viewed it as a distraction from the stress or as therapeutic. Thus, any future trial would need to consider the writing ability and experiences of caregivers. In fact, the ability to emotionally express in writing exercises has been found to be key moderating factor in these studies (Niles, Haltom, Mulvenna, Lieberman, & Stanton, 2014). Finally, we also checked whether our dropouts in each group differed across all the parameters discussed earlier, as well as sociodemographic, to see if one group was more vulnerable to dropout than the other. The only difference observed was that those in the control condition who dropped out were more anxious at baseline and were slightly older, albeit these were not statistically different, *p* = .05 and *p* = .07, respectively. There were no differences across all the other key sociodemographic, caring, intervention, or psychosocial variables. However, while we recruited our target sample size, these were not maintained at follow‐up, and thus our findings must be interpreted with caution as we were likely underpowered to detect effects, and future studies should aim to improve retention.

### Limitations

Besides the methodological concerns identified above, several other limitations must be acknowledged. Although in the present study techniques were adopted to reduce attrition and improve adherence (financial incentive, reminder emails) there was a high attrition rate (48.9%) at final follow‐up and a final sample size of 45 caregivers. Although this is a weakness in the current trial, high attrition rates and dropouts from Internet‐based trials are regularly substantial (Wantland, Portillo, Holzemer, Slaughter, & McGhee, 2004) and are a common feature in similar intervention studies (Bolier et al., 2013). Given the unique nature of the caregiver population, that is, limited personal time and self‐care, time constraints, difficulty in accessing research, and mistrust of researchers (Gallagher‐Thompson et al., 2003; Lampley‐Dallas, 2002; Moreno‐John et al., 2004; Pasacreta & McCorkle, 1999), the current sample size is not unusual when compared to other expressive writing studies (Lovell et al., 2016). It is also possible that many participants dropped out because the intervention was not producing significant benefits and as such there may have been little intrinsic motivation to continue and complete all follow‐ups. Thus, to address these biases, sensitivity analysis using multilevel modelling was undertaken and yielded similar results to the complete case analysis. Additionally, in a desire to give the participants a sense of confidentiality and meet the needs of the caregivers in our study, it was not mandatory that diaries be returned, and instead self‐reported adherence was relied on. As such, there is a level of uncertainty with regard to participant compliance and treatment integrity (Weck, Bohn, Ginzburg, & Stangier, 2011), which also necessitates cautious interpretation of the null findings. In future trials, this would be better accessed through the use of timestamps, when interventions are completed online electronically or through websites (Krejtz, Nezlek, Michnicka, Holas, & Rusanowska, 2016). Further, if a full‐scale trial were to be conducted, and based on a recent meta‐analyses of small effect sizes for both writing interventions and positive psychology interventions which this study is more akin to (e.g. Davis et al., 2016; Pavlacic, Buchanan, Maxwell, Hopke, & Schulenberg, 2019), a sample size of 280 would be needed. Moreover, researchers would also need to consider the risk of high attrition and perhaps think about ways to minimise this with some of the suggestions above. Taken together, while we did not observe any changes on our outcome variables from this 2‐week intervention, it was feasible and acceptable to our caregiver groups. However, while it was feasible, based on the qualitative comments it may not be suitable for all carers, especially those who may struggle with expressing themselves through writing. Further, this also suggests that any future BF writing intervention would require several procedure modifications including tailoring to a specific cohort of caregivers (in particular those who like writing), having a longer duration and spacing of the writing intervention, altering the neutral condition and consideration of other moderators (e.g. social support). In fact, the qualitative comments show that there were some benefits reported, including reappraisal and distraction from the stressor, which are well‐established coping strategies. Finally, given that caregivers are constrained for time and accessibility to research sites (Dowling & Wiener, 1997; Gallagher‐Thompson et al., 2003; Lampley‐Dallas, 2002), these type of interventions may, when tailored and modified, have some utility.

## Competing Interests

The authors declare that they have no competing interests.
